# 8806 Russian patients demonstrate T cell count as better marker of COVID-19 clinical course severity than SARS-CoV-2 viral load

**DOI:** 10.1038/s41598-021-88714-6

**Published:** 2021-05-03

**Authors:** Konstantin S. Sharov

**Affiliations:** Koltzov Institute of Developmental Biology, Russian Academy of Sciences, 26 Vavilov Street, Moscow, Russia 119334

**Keywords:** Infectious diseases, Signs and symptoms

## Abstract

The article presents a comparative analysis of SARS-CoV-2 viral load (VL), T lymphocyte count and respiratory index PaO_2_:FiO_2_ ratio as prospective markers of COVID-19 course severity and prognosis. 8806 patients and asymptomatic carriers were investigated in time interval 15 March–19 December 2020. T cell count demonstrated better applicability as a marker of aggravating COVID-19 clinical course and unfavourable disease prognosis than SARS-CoV-2 VL or PaO_2_:FiO_2_ ratio taken alone. Using T cell count in clinical practice may provide an opportunity of early prediction of deteriorating a patient’s state.

## Introduction

There is a definite need in laboratory markers of COVID-19 clinical course severity and prediction of the disease outcome. Several COVID-19 prognosis predicting models have been proposed lately^1–7^. Most of them are one-factor, but there are complex multifactor models too. Skevaki et al.^8^ and Galloway et al.^9^ used data on severe COVID-19 patients, trying to identify the major laboratory markers of very serious cases and mortality. In these studies, the data obtained on laboratory markers were rather contradictory. Most importantly, the size of sample sets was restricted (several dozen or several hundred patients at the utmost). Limited volume of experimental data may have been the main reason of many models’ low accuracy observed hitherto.

Currently SARS-CoV-2 viral load (VL) is almost universally regarded as a factor directly related to COVID-19 severity and prognosis and, therefore, one of the major indicators of the disease progression^10–14^. However, recently it has been demonstrated by a number of research groups that it may be an oversimplified, if not incorrect approach^15–20^. According to the recent findings, VL may indicate some COVID-19 severe cases such as “cytokine storm” or serious disease progression in immunosuppressed patients but is problematic to apply as a marker of COVID-19 severity universally^21–24^. For VL to become a reliable marker of COVID-19 severity, in most cases it must be supplemented with other markers, as merely one parameter can be hardly used for a satisfactory description of such complex system as a full set of COVID-19 clinical manifestation^25–27^. We think that T-cell immune response may be a good choice.

We report our findings in studying interdependence of

1. SARS-CoV-2 VL,
2. Leukocyte, B and T lymphocyte count,
3. Respiratory marker PaO_2_:FiO_2_ ratio

in COVID-19 patients/carriers to find the most demonstrative laboratory markers of COVID-19 clinical course severity and prognosis.

## Methods

### Patients and clinical data

8806 patients/carriers were investigated since 15 March to 19 December 2020, whose SARS-CoV-2 positive status was initially proven by molecular biology. Objective and subjective information about COVID-19 clinical manifestations was recorded in hospitals and clinics in twelve Russian regions (Moscow, Moscow region, St Petersburg, Nizhny Novgorod, Murmansk, Dagestan, Komi, Krasnoyarsk, Tyumen, Krasnodar, Khabarovsk, and Vladivostok).

Twenty-six hospitals, thirty-four outpatients’ clinics and ambulance centres, and sixteen non-commercial test labs and medical centres were involved in the research. Symptomatic patients and asymptomatic carriers were included in the group. The patient sample set composition gives an opportunity to regard the set as random. Age, gender, results of general clinical blood tests, X-ray CT results, clinical symptomatic picture (or the fact of symptoms absence for asymptomatic carriers), time of symptom onset, clinical procedures including admission to general wards and ICU as well as outcome were known. No patient identifying information was known or disclosed.

### Measurements

RT-qPCR technique was applied to make SARS-CoV-2 VL measurements (Bio-Rad CFX Automation System II, Hercules, CA, USA+ Vector Multiplex RT-qPCR SARS-CoV-2 Test Kit, Novosibirsk, Russia). The nasopharyngeal or oropharyngeal swabs were taken in clinics/hospitals or at home in case of asymptomatic or mild symptomatic carriers and COVID-19 out-patients. Flow immunofluorocytometry (MACSQuant Analyzer 16 Flow Cytometer, Miltenyi Biotec, Bergisch Gladbach, Germany) with necessary Miltenyi Biotec and Vector reagents and kits was used for performing immunological cell blood analyses. For isolation of T killer cells, MACSxpress CD8 T Cell Isolation Kit was used + specific marker presence/absence check during flow cytometry CD27– CD28– CD45RA+ Perforin+; for Th1 helpers MACSxpress CD4 T Cell Isolation Kit + IFN-γ Secretion Assay—Cell Enrichment and Detection Kit; for isolation of γδT cells Anti-TCRγ/δ MicroBead Kit; for NKT cells Anti-iNKT MicroBeads; for CD8αα+ cells Vector CD8 homodimer Isolation Kit; for B1 cells Miltenyi Biotec StraightFrom Whole Blood CD19 MicroBead Kit, StraightFrom Whole Blood CD45 MicroBead Kit + CD20 and CD43 markers presence on cytometry; for plasma cells Miltenyi Biotec StraightFrom Whole Blood CD138 MicroBeads.

For symptomatic patients, VL was measured in nasopharyngeal/oropharyngeal swabs taken on Days 1–4 since symptomatic onset. The day of onset was taken as Day Zero. According to the literature^28–33^, VL is maximal approximately at this time, for COVID-19 symptomatic clinical course. For asymptomatic carriers, if a test for SARS-CoV-2 RNA was positive and the person in question expressed his/her wish to participate in the research, the day of test was conventionally counted as Day Zero and subsequent days received corresponding numbers. Total leukocyte count and B1 cell counts were measured in blood samples taken on Days 2–4. Plasma cell and T cell counts were measured on Days 8–12 in serum and pharyngeal mucosa-associated lymphoid tissue (MALT). As we found in another study of a relatively small group of HIV/SARS-CoV-2 co-infected people, the maximum of T cell response to SARS-CoV-2 acute infection is usually observed since Day 6 to 16^34^. As well, PaO_2_/FiO_2_ ratio was measured on Days 8–12 (1) to correspond roughly with T cell measurements; and (2) since it was demonstrated by different groups of researchers that severe COVID-19-associated ARDS is generally detected after the first week of the COVID-19 disease^35–38^. Two separate intakes and two separate measurements with calculating the average value were made in every analysis, to reduce the contribution of experimental errors.

### Subjective symptomatic score

We introduced a so-called “subjective symptomatic score” whose invention was partly inspired by methodology described in works of Calza et al.^39^ and Galloway et al.^9^. However, we used our own modification of this factor. The presence of every symptom has a score of + 1 – + 5 depending on the pronouncedness, its absence 0. Medical personnel was consulted to apply such a system of evaluation and we received the numerical data in the majority of cases. Where numerical data were missing, we transformed qualitative description of symptomatic course into the score discussed, by ourselves. While the symptomatic picture is definitely subjective as it is based on personal perception of COVID-19 symptoms or observations of medical staff and therefore qualitative, the score may give a semi-quantitative indication of COVID-19 course severity. The list of symptoms taken into account are summarized in Table 1.

**Table 1:** Symptoms of COVID-19 taken into account for calculating subjective symptomatic score (the indicator showing subjective perception of the disease by a patient or qualitative clinical observations). Subjective symptomatic score runs from 0 to 100.

No	Symptom
1	Fever
2	Cough
3	Myalgia
4	Headache
5	Anosmia
6	Ageusia
7	Perspiration/hyperhidrosis
8	Fatigue
9	Dyspnoea
10	Rhinitis
11	Pharyngitis
12	Vertigo
13	Sneezing
14	Anorexia
15	Nausea
16	Vomiting
17	Abdominal pain
18	Diarrhoea
19	Haemoptysis
20	Fear/panic attacks/mental confusion

As one can conclude, asymptomatic carriers had the total score of 0. The maximal possible value is 5 × 20 = 100. When calculating subjective symptomatic score for symptomatic cohort, we did not count asymptomatic values lest the score should be greatly skewed to low values.

### Acute respiratory distress syndrome (ARDS) score

We suggest using ARDS score *AS* to assess COVID-19 respiratory complications. We may define it as

$$AS = \frac{1000}{{{\text{PaO}}_{2} :{\text{FiO}}_{2} {\text{ ratio}}}} + \left[ {100 - {\text{SpO}}_{2} } \right] + \left[ {RR - 20} \right],$$

where *RR* is respiratory rate [min^–1^]. The detailed description of this indicator is provided in our another work^34^. An advantage of using *AS* over any of PaO_2_/FiO_2_ ratio, SpO_2_ or *RR* can be explained by the complex nature of *AS*. *AS* encompasses more border states of ARDS than any of these three indicators. Therefore, in some cases using *AS* may be more reliable as it usually highlights suspicious cases of ARDS that some of the three separate indicators may miss.

The formula for *AS* contains reversed PaO_2_/FiO_2_ ratio as the first item (with multiplier 1000 taken for convenience). It explains why we used reversed PaO_2_/FiO_2_ ratio in the current study instead of PaO_2_/FiO_2_ ratio that is generally used as an indicator of respiratory state of a patient. Indeed, we searched for direct proportionality between VL and COVID-19 respiratory complications. One can expect that high VLs may potentially indicate severe respiratory failure, as it was shown in numerous research papers, e.g. in the works^40–42^. Low PaO_2_/FiO_2_ ratio values which stand for respiratory failure do not correlate with high VLs as direct proportionality, but high reversed PaO_2_/FiO_2_ ratios do. However, using reversed PaO_2_/FiO_2_ ratio was a mere convention dictated by the simplicity of formulas in the current study. It can be easily substituted by PaO_2_/FiO_2_ ratio.

### Statistical analysis and visualisation

OriginPro ver. 9.2.196 (OriginLab Corporation, Northampton, Mass., USA, https://www.originlab.com) was used for statistical calculations and visualisation.

### Type of study

The study does not represent a randomised controlled clinical trial. All relevant measurements and data processing were performed retrospectively. Reporting of the study conforms to broad EQUATOR guidelines.

### Informed consent for participation and publication

An informed consent has been given by the patients for using their anonymised clinical data for scientific investigations and publication. It was duly signed and kept in the respective medical institutions. For minor human participants under the age of 18 years, informed consent has been obtained from their parent and/or legal guardian.

### Ethics guidelines

The Ethical Committee of Koltzov Institute of Developmental Biology of Russian Academy of Sciences regarded the current study ethically appropriate and exempt from human subjects review, as (1) no private identifying information was known and, consequently, disclosed by the authors; (2) all experiments were performed in accordance with relevant guidelines and regulations; and (3) all the methods used were approved as conforming to the relevant guidelines and regulations (permission no. 39100920).

### Ethics approval

Granted by Ethical Committee of Koltzov Institute of Developmental Biology of Russian Academy of Sciences (no. 39100920).

## Results and discussion

### Main clinical parameters

Demographics and main clinical parameters of the patients are summarised in Table 2.

**Table 2:** Demographics of the patients studied and their main clinical parameters.

Age	Range 15–94 years; mean 52.2 ± 13.7 years (CI 95%, *p* = 0.05)		
Gender	4526 females (51.4%)		
Most frequent comorbidities	*Comorbidity*		*Number of patients documented*
	Following bad habits (chronic smoking, alcoholism, drug addiction)		2301 (26.1%)
	Metabolism disorders (diabetes, obesity, etc.)		1876 (21.3%)
	Cardiovascular diseases (hypertension, coronary artery disease, etc.)		1742 (19.8%)
	Internal organ diseases (COPD, asthma, interstitial lung disease, chronic kidney disease, etc.)		361 (4.1%)
	Oncological diseases		158 (1.8%)
	Functional impairments, including immune system disorders		114 (1.3%)
	Chronic infectious diseases, including HIV and hepatitis viruses induced diseases		79 (0.9%)
	Neurological diseases		27 (0.3%)
Most frequent complications	*Complication*		*Number of patients documented*
	Viral pneumonia (COVID-19- only or COVID-19- + another viral causative agent related pneumonia) with the cut-off threshold 10% of lung CT images. Patients with less expressed CT opaqueness were not counted		634 (7.2%)
	Secondary bacterial pneumonia		247 (39.0% of all patients with COVID-19-related pneumonia)
	Pulmonary embolism		48 (0.5%)
	Internal organ malfunction		11 (0.1%)
	“Cytokine storm,” i.e. severe and prolonged hyperinflammation associated with elevated blood concentration of inflammatory cytokines		37 (0.4%)
COVID-19 clinical course classification	*Clinical course*		*Number of patients documented*
	Asymptomatic		6211 (70.5%)
	Mild		1285 (14.6%)
	Moderate		732 (8.3%)
	Pronounced (severe)		486 (5.5%)
	Critical		92 (1.1%)
Mode of treatment	*Predominant place of stay*		*Number and percentage in respective cohort*
	Outpatients		7875 (89.4%)
	Hospital stay (inpatients)		931 (10.6%)
	ICU treatment with non-invasive oxygenation		111 (11.9% of all hospital patients)
	ICU treatment with mechanical ventilation		44 (21.8% of all ICU-admitted patients)
	ICU treatment with ECMO		47 (23.3% of all ICU-admitted patients)
Clinical outcome	*Outcome*		*Number and percentage of respective cohort*
	Mortality		71 (0.8% of all set; 7.6% of hospital inpatients; 35.1% of ICU patients)
	Full recovery with positive coenesthesia (subjective health perception) after discharge from hospital		182 (22.0% of all patients discharged from hospitals)
	Recovery with pulmonary fibrosis		53 (8.4% of all COVID-19-related pneumonias)
	Recovery with prolonged negative coenesthesia after discharge from hospital		644 (78.0% of all patients discharged from hospitals)
Mean body temperature of hospital patients, measured in the evening (mainly at 5 pm–11 pm)	*Days since symptomatic onset*		*Body temperature, °C (CI 95%, p* = *0.05)*
	1st day		37.7 ± 0.8
	2nd day		37.9 ± 0.9
	3rd day		37.6 ± 1.0
	7th day		37.2 ± 0.5
	14th day		36.3 ± 0.6
	21st day		36.5 ± 0.7
Lung CT opaqueness, per cent	*Type of patient*		*Percentage of lung tissue with interstitial pneumonia picture (CI 95%, p* = *0.05)*
	ICU hospital patients		57.3 ± 24.8%
	Non-ICU hospital patients		32.4 ± 18.3%
	Outpatients		18.5 ± 12.7%
COVID-19 severity	Subjective symptomatic score	Outpatients, excluding asymptomatic carriers	21 ± 15
		Inpatients	48 ± 22
	ARDS score *AS*	Outpatients, excluding asymptomatic carriers	1.7 ± 0. 5
		Inpatients	5.6 ± 3.1

### SARS-CoV-2 viral load as prospective marker of COVID-19 course severity

**Figure 1:**
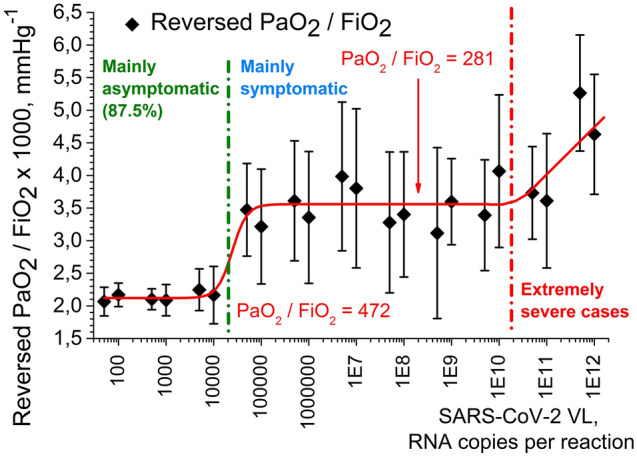
Correlation between reversed PaO_2_/FiO_2_ ratio as an indicator of COVID-19 respiratory complications severity (the first item in ARDS score formula) and SARS-CoV-2 VL. In this and subsequent figures Confidence interval (CI) is 95%. Standard error of mean is showed as whiskers. Green dash-dotted line stands for the conditional border between asymptomatic carriers and symptomatic patients. Red dash-dotted line stands for the conditional border between extremely severe clinical cases and the rest of the patients. Percentage numbers in parentheses (green) correspond to the proportion of asymptomatic carriers whose manifestations fit into the conditional range to the left of the green line. Created in OriginPro ver. 9.2.196, https://www.originlab.com.

Figure 1 demonstrates relationship between SARS-CoV-2 VL and reversed PaO_2_/FiO_2_ ratio (the first item in the formula for ARDS score *AS*, see “Methods” section).

We can observe that there is no clear correlation between reversed PaO_2_/FiO_2_ ratio and VL for the whole range of VLs. None the less, the almost linear piecewise dependence of reversed PaO_2_/FiO_2_ ratio on SARS-CoV-2 VL (several linear fragments) helps to isolate asymptomatic carriers (the leftmost points in Fig. 1) and severest cases (the rightmost points), whereas the majority of VLs corresponds to approximately the same reversed PaO_2_/FiO_2_ ratio = 3.6 ± 1.3 mmHg^–1^ (horizontal plateau in the middle of Fig. 1). That corresponds to direct PaO_2_/FiO_2_ ratio range = [204…434 mmHg].

Statistical analysis gave the following results. For reversed PaO_2_/FiO_2_ ratio dependence on VL (Fig. 1), approximation function was logistic function of accumulation (sigmoidal):

$$\frac{1000}{{{\text{PaO}}_{2} { }:{\text{ FiO}}_{2} }}\left[ {{\text{mmHg}}^{ - 1} } \right] = A_{2} + \frac{{A_{1} - A_{2} }}{{1 + \left( {\frac{VL}{{VL_{0} }}} \right)^{p} }},$$

with coefficients: *A*_1_ = 2.1200, *A*_1_ (SE—henceforward Standard Error of Mean) = 0.1188 [mmHg^–1^]; *A*_2_ = 3.5585, *A*_2_ (SE) = 0.0718 [mmHg^–1^]; *VL*_0_ = 24,532, *VL*_0_ (SE) = 14,032 [RNA copies per reaction]; *p* = 2.8127, *p* (SE) = 1.9974. The statistical analysis parameters: *χ*_*red.*_^2^ = 0.0646; *R*_*adj.*_^2^ = 0.8688, Fisher coefficient *F* = 787.77.

In Fig. 1 we see that only very high VL led to the distortion of plateau PaO_2_/FiO_2_ = 281 mmHg (to the right of the red vertical line, i.e. further diminishing PaO_2_/FiO_2_), whereas the majority of VLs were statistically undistinguishable in terms of PaO_2_/FiO_2_ ratio.

### Leukocyte and B lymphocyte counts in serum as prospective markers of COVID-19 course severity

**Figure 2:**
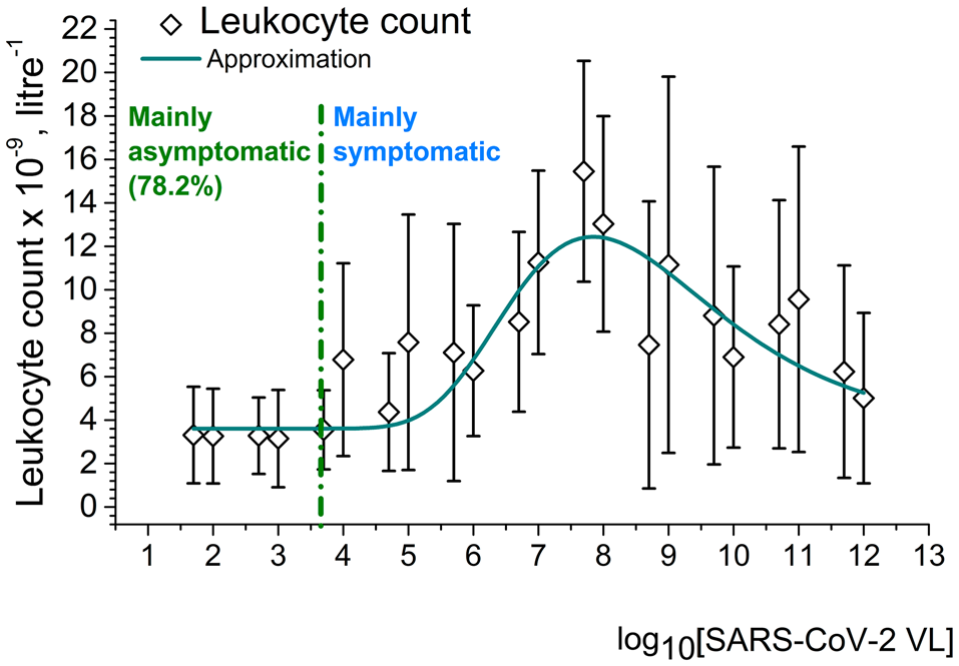
Correlation between leukocyte count in serum on logarithm of SARS-CoV-2 VL. Created in OriginPro ver. 9.2.196, https://www.originlab.com.

**Figure 3:**
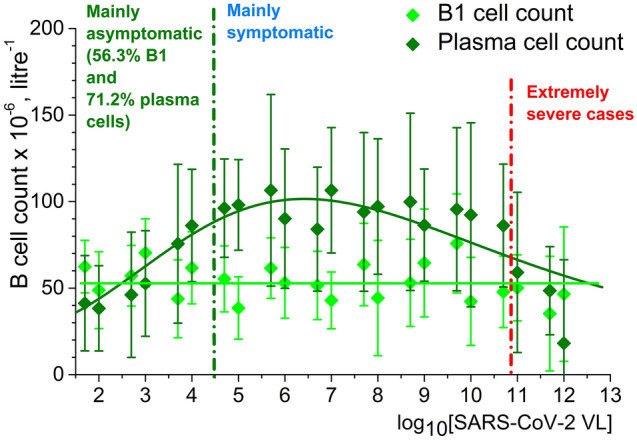
Correlation between B1 cell (light green line) and plasma cell (dark green line) counts in serum on logarithm of SARS-CoV-2 VL. Created in OriginPro ver. 9.2.196, https://www.originlab.com.

In Fig. 2 one can see the dependence of total leukocyte count in serum on logarithm of SARS-CoV-2 VL and in Fig. 3 the dependence of B lymphocyte subpopulation counts on logarithm of SARS-CoV-2 VL. Leukocyte count (more exactly, granulocyte count) may be understood as a common indicator of the front line of innate immune system response to SARS-CoV-2 and B cell counts as parts of both innate and adaptive immune response. However, again we do not observe any direct proportionality between VL and overall white blood cell counts, nor B lymphocyte counts, as the correlations are non-linear or there are no correlation at all (for B1 cells, Fig. 3).

Both leukocyte and plasma cell count correlations with SARS-CoV-2 VL have an asymmetric wave shape with maximums around VLs of 10^5^–10^8^ RNA equivalents per reaction. These maximums correspond with the majority of symptomatic patients. In asymptomatic carriers, immune response is not pronounced and almost all severest cases were cases of patients with chronic immune problems. This may indicate that the strongest immune response (innate and adaptive) to SARS-CoV-2 is present in COVID-19 symptomatic or at least paucisymptomatic patients without serious immune disorders.

Therefore, we may suggest that neither overall white blood cell count, nor B cell count can serve as an evident marker of COVID-19 clinical course severity.

### Viral load and severe clinical cases

**Figure 4:**
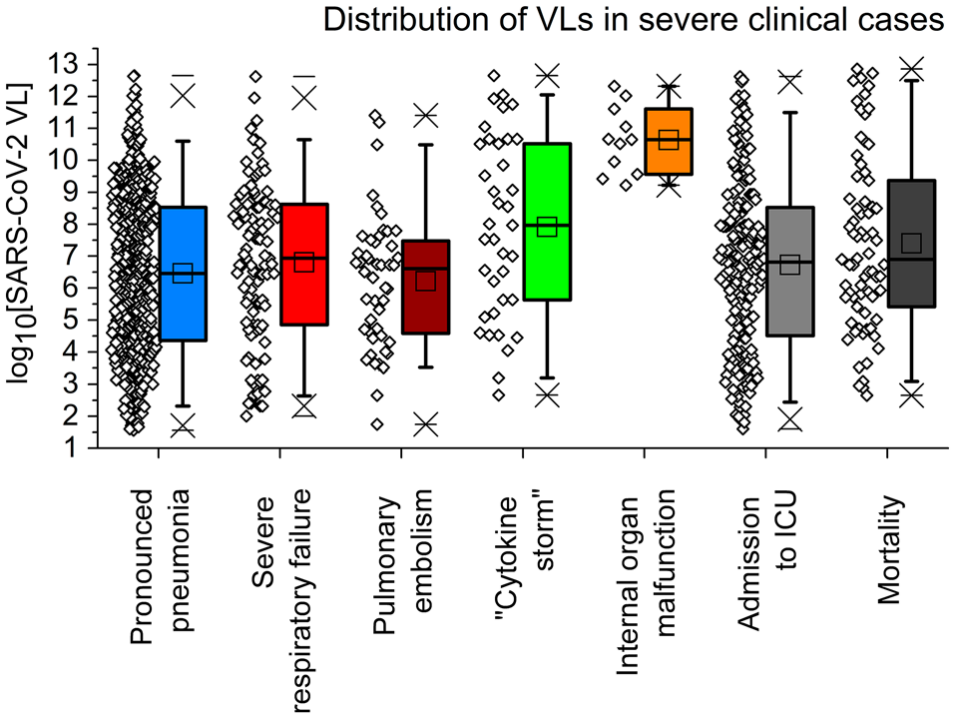
Distribution of SARS-CoV-2 VLs in severe clinical cases: (1) serious viral interstitial pneumonia with X-ray computer tomography opaqueness not less than 15% (469 cases, 5.33%); (2) severe respiratory failure of type 1 [either of PaO_2_/FiO_2_ ratio < 280 mmHg OR respiratory rate  > 30 min^–1^ OR SpO_2_ < 90% (ARDS score *AS* > 10–12)] (103 cases, 1.17%); (3) pulmonary embolism (48 cases, 0.55%); (4) immune response dysregulation known as “cytokine storm” (37 cases, 0.42%); (5) internal organ malfunction (11 cases, 0.12%); (6) transfer from general ward to ICU (202 cases, 2.29%); (7) lethal outcome (71 cases, 0.81%). Individual cases and statistical boxes are visualised along each other. Boxes: 25/50/75%. Whiskers: 5/95%. Horizontal lines are maximums/minimums, crosses stand for 1/99%. Created in OriginPro ver. 9.2.196, https://www.originlab.com.

In the works^24,43,44^ it was assumed that SARS-CoV-2 VL may be an indicator of COVID-19 course severity in specific disease cases, e.g. in the severest cases or complications. We suggested that VL might correlate with the most serious cases. In Fig. 4, we plot the distribution of VLs in the severest COVID-19 patients.

A careful look at Fig. 4 proves that SARS-CoV-2 VL cannot be used as an unequivocal marker of COVID-19 complications, excepting internal organ malfunction (orange box). Very broad ranges of VLs correspond with COVID-19 serious complications (pronounced interstitial pneumonia, respiratory failure, acute respiratory distress syndrome (ARDS), pulmonary embolism, “cytokine storm,” etc.).

### T lymphocyte count as prospective marker of COVID-19 course severity

**Figure 5:**
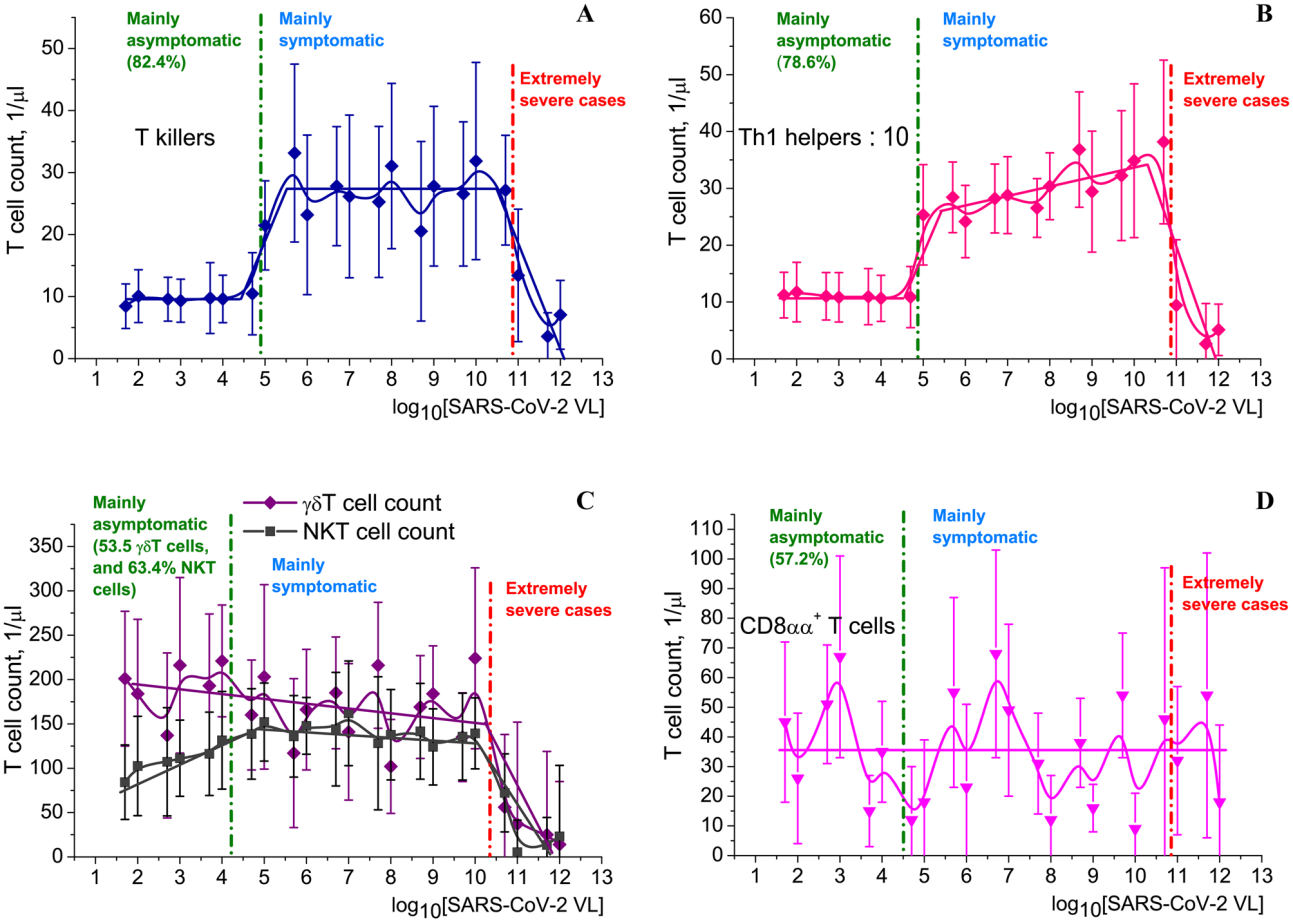
T cell count dependence on logarithm of SARS-CoV-2 VL. Piecewise and smooth approximations are shown. Green dash-dotted line stands for the conditional border between asymptomatic carriers and symptomatic patients. Red dash-dotted line stands for the conditional border between extremely severe clinical cases and the rest of the patients. Percentage numbers in parentheses (green) correspond to the proportion of asymptomatic carriers whose manifestations fit into the conditional range to the left of the green line. (**A**) T killer (CD8^+^ cytotoxic lymphocyte) count (in serum) dependency on log_10_VL. (**B**) Th1 helper (CD4^+^ CD94 + lymphocyte) count (in serum) dependency on log_10_VL. (**C**) γδT and NKT lymphocyte counts (in serum) dependency on log_10_VL. (**D**) CD8αα^+^ lymphocyte count (in pharynx MALT analysed in nasopharyngeal or oropharyngeal swabs) dependency on log_10_VL. Created in OriginPro ver. 9.2.196, https://www.originlab.com.

**Figure 6:**
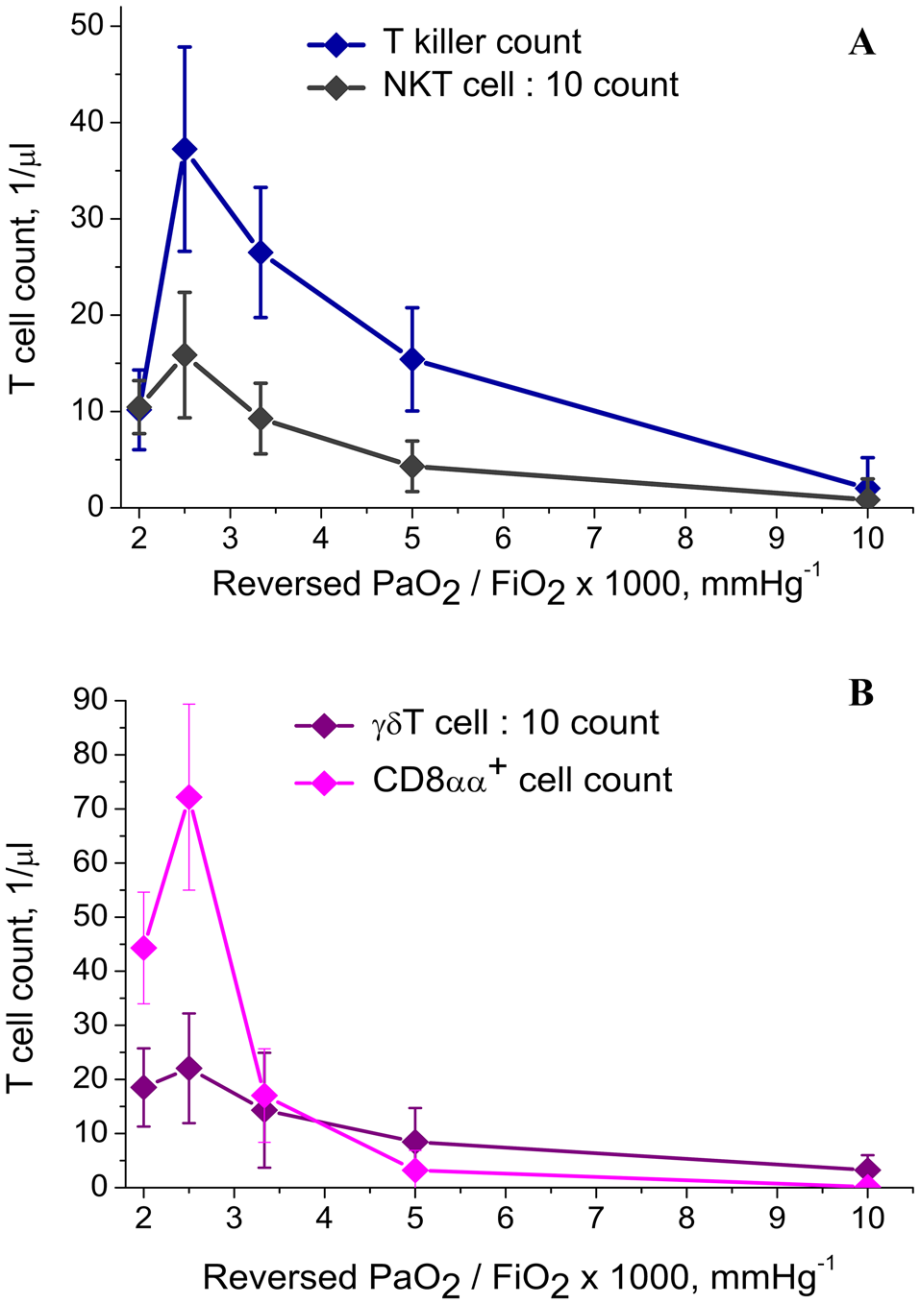
T cell count dependence on respiratory factor reversed PaO_2_/FiO_2_ ratio. Linear piecewise approximation is shown. (**A**) T killer and NKT cell counts dependency on PaO_2_/FiO_2_ ratio. (**B**) γδT and CD8αα+ cell counts dependency on PaO_2_/FiO_2_ ratio. Created in OriginPro ver. 9.2.196, https://www.originlab.com.

To find such markers, we studied correlation between (1) VL and T lymphocyte counts (Fig. 5); and (2) reversed PaO_2_/FiO_2_ ratio and T lymphocyte counts (Fig. 6).

Dependence of T killer (cytotoxic CD8^+^ T lymphocyte) [membrane phenotype CD3^+^ CD8^+^ CD45RA^+^ CD27^–^ CD28^–^ CCR7^–^ CD178(FasL)^+^ CD107a(LAMP-1)^+^ IFNγ^+^ Granzyme B^+^ Perforin-1^+^] count (Fig. 5A) and Th1 helper [membrane phenotype CD3^+^ CD4^+^ CD94 + CD183(CXCR3)^+^ CD195(CCR5)^+^ CCR3^–^ CCR4^–^ CXCR4^–^] count/10 (one tenth part) (Fig. 5B) in serum on logarithm of SARS-CoV-2 VL provides a promising instrument for marking very serious and aggravating COVID-19 clinical cases. Correlation between T killer and Th1 helper counts was very strong (Pearson correlation coefficient *C* = 0.91371, *p* = 0.01258).

Dependence of γδT [membrane phenotype CD3^+^ TCRγ/δ^+^ IFNγ^+^] and NKT [membrane phenotype CD3^+^ CD161(NK1.1/NKR-P1)^+^ CD16^+^ CD56^+^ CD57^+^ CD44^+^ CD69^+^] lymphocyte count on logarithm of SARS-CoV-2 VL (Fig. 5C) demonstrates the absence of statistically significant dependence of cell count on VLs for asymptomatic/symptomatic patients/carriers distinguishing. For extremely severe (critical) cases, a strict diminishment was observed. It may be an indication that SARS-CoV-2 influence on immunity is related to mainly adaptive immunity, as NKT cell selection and homeostasis are connected with adaptive immunity regulation, whereas γδT cells are a part of innate immunity.

Distribution of CD8αα^+^ cells on VL is presented in Fig. 5D. Homodimeric CD8αα^+^ cells are a “non-classical” subpopulation of γδT-lymphocytes whose membrane phenotype may be described as CD3^+^ CD8αα^+^ CD2^–^ CD5^–^ CD28^–^ CD4^+/–^^45,46^. Instead of dimer ζ_2_, in their TCR they contain homodimer FcεRIγ/FcεRIγ or heterodimer ζ/FcεRIγ. Homodimer CD8αα does not act as a co-receptor—otherwise CD8αα^+^ cells could recognise an antigen presented within MHC-I, and CD8αα^+^ response to antigen is likely to proceed with “non-classical” MHC-I molecules, probably along Qa or TL pathways.

In Fig. 6, dependence of T lymphocyte population size on reversed PaO_2_/FiO_2_ ratio is presented.

Here the dependence is much stronger than the dependence on VL. Statistically significant difference in T killer, NKT cell (Fig. 6A), γδT and CD8αα^+^ cell population size (Fig. 6B) gives an opportunity to use this functional relation as a more unequivocal marker of COVID-19 course severity than T cell count—VL dependency. We did not detect any connection of CD8αα^+^ cell count on SARS-CoV-2 VL (Fig. 5D), but observed strong diminishment of CD8αα^+^ lymphocyte population size in pharynx MALT with the growth of respiratory distress severity (Fig. 6B).

### Markers of clinical course outcome, mortality and prognosis

To find a convincing marker of COVID-19 clinical course outcome, including mortality, may be regarded a separate important task in suggesting markers for COVID-19 clinical course predictions.

COVID-19-related mortality was closely connected with T cell population suppression in severest cases. T cell population size diminishment (especially of T killers, Th1 helpers, and CD8αα^+^ lymphocytes) was more closely connected with COVID-19-related mortality (Pearson correlation coefficient *C* = 0.7411 at *p* = 0.1217) than PaO_2_/FiO_2_ ratio with COVID-19-related mortality (*C* = 0.6238 at *p* = 0.2150). But even more importantly, as we detected, strong diminution of T cell populations often (in 58.22% cases) shows deterioration of a patient’s conditions 2–5 days before worsening his/her respiratory status measured by PaO_2_/FiO_2_ ratio.

**Figure 7:**
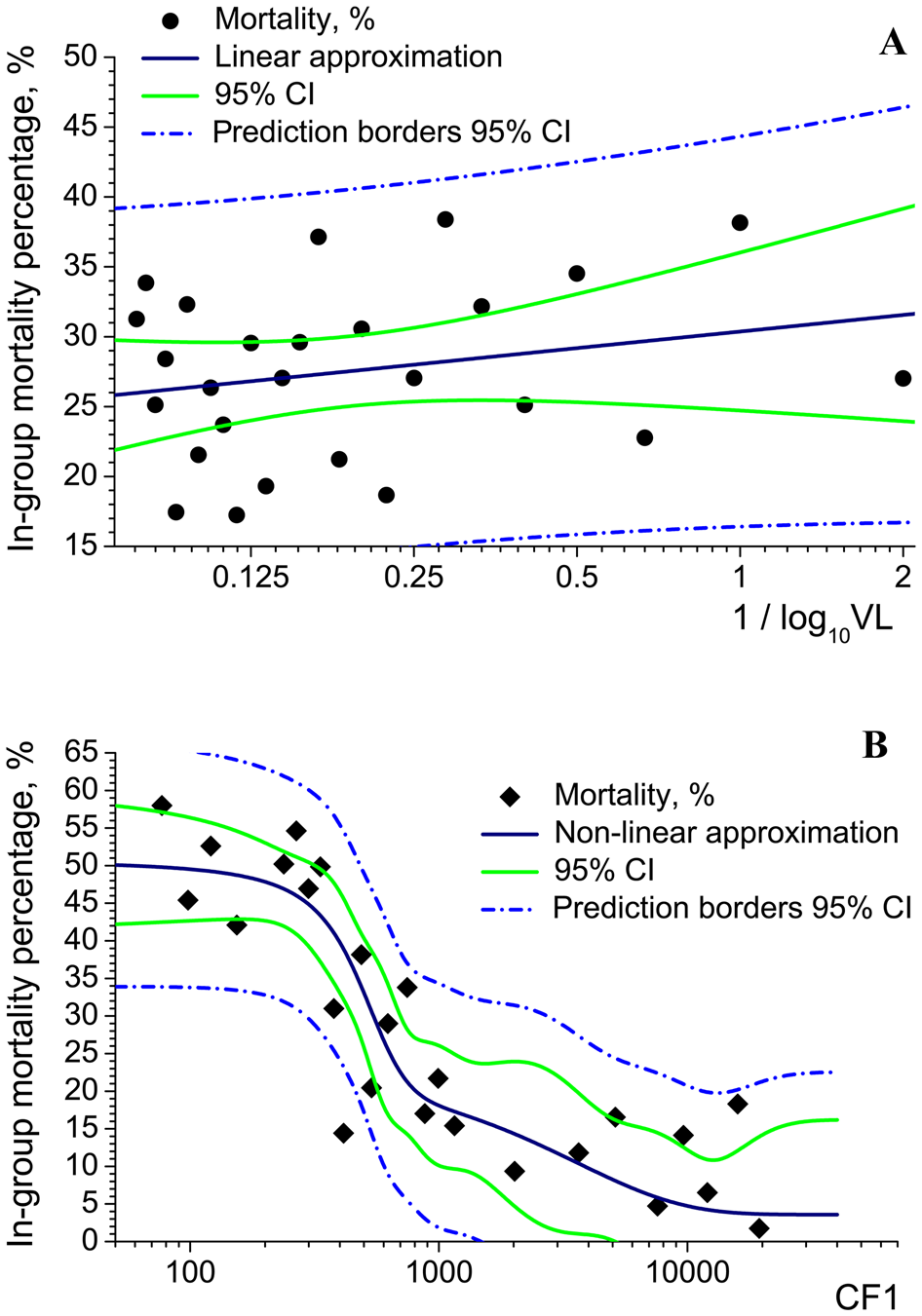
Dependence of in-group mortality percentage on VL (**A**) and CF1 (**B**), experimental data and approximation. For detailed description please see the text. Created in OriginPro ver. 9.2.196, https://www.originlab.com.

The mortality distribution on VLs (Fig. 4, rightmost dark-grey box) did not demonstrate direct proportionality relationship between VL (or its reversed logarithm $\frac{1}{{\log_{10} {\text{VL}}}}$) and lethal outcome. While mortality net number was distributed in an asymmetric peak-like mode, not Gaussian mode, the distribution of mortality percentage did not show any clear dependence on VL (Fig. 7A). The approximation dependence

$$Mortality, \% = a + b \cdot \log_{2} \left( {\frac{1}{{\log_{10} {\text{VL}}}}} \right) = a + b \cdot \log_{2} \left( {\log_{{{\text{VL}}}} 10} \right)$$

is very weak and even unreliable, that can be observed from the poor approximation parameters values: *a* = 26.82, *a* (SE) = 1.53; *b* = 2.39, *b* (SE) = 3.08; Fisher coefficient *F* = 0.6030; Pearson’s *r* coefficient = 0.1566; *R*_*adj.*_^2^ = –0.0161. The prediction force is low and, for severest COVID-19 cases, mortality percentage prediction deviates enormously from 8 to 40%.

However, the mortality percentage distribution on the product of several factors (“complex factor” CF1)

$$\begin{aligned} {\text{CF}}1 & = \frac{1}{{\log_{10} {\text{VL}}}} \times {\text{T}}\;{\text{killer}}\;{\text{count}} \times {\text{Plasma}}\;{\text{cell}}\;{\text{count}} \times {\text{NKT}}\;{\text{cell}}\;{\text{count}} \\ & = \log_{{{\text{VL}}}} 10 \times {\text{T }}\;{\text{killer}}\;{\text{count}} \times {\text{Plasma}}\;{\text{cell}}\;{\text{count}} \times {\text{NKT}}\;{\text{cell}}\;{\text{count}} \\ \end{aligned}$$

did demonstrate a stronger dependence (Fig. 7B). The best fit can be achieved through approximating by biphasic dose–response function:

$$Mortality,\% = A_{1} + \left( {A_{2} - A_{1} } \right)\left( {\frac{p}{{1 + 10^{{\left( {\log_{10} x_{1} - \log_{10} {\text{CF}}1} \right)h_{1} }} }} - \frac{1 - p}{{1 + 10^{{\left( {\log_{10} x_{2} - \log_{10} {\text{CF}}1} \right)h_{2} }} }}} \right),$$

*A*_1_ = 3.58, *A*_1_ (SE) = 2.38; *A*_2_ = 3906.18, *A*_2_ (SE) = 1380.15; log_10_ *x*_1_ = 482.38, log_10_ *x*_1_ (SE) = 55.14; log_10_ *x*_2_ = –19,014.35, log_10_ *x*_2_ (SE) = 4639.34; *h*_1_ = –0.0040; *h*_1_ (SE) = 0.0027; *h*_2_ = –0.000121; *h*_2_ (SE) = 0.000397; *p* = 0.00729; *p* (SE) = 0.00925.

By comparing results of the statistical analyses, we see that the complex factor CF1 is more promising for using as an input variable for predicting lethal outcome than VL. In the defining formula for CF1, we did not include Th1 helper count, as Th1 count was shown to be very strongly correlated with T killer count (Fig. 5A,B). Therefore, of the two variables T killer and Th1 helper counts, a truly independent variable is merely one.

The approximation by biphasic dose–response function is still imperfect and 95% confidence interval boundaries are very wide (Fig. 7B). E.g., for COVID-19 severest clinical course (CF1 < 300) μl^–3^, prediction range for mortality percentage spans from 34 to 66%. However, this approximation provides one of the most precise predictive ranges for mortality.

### Limitations of the study

The research has serious limitations that cannot be ignored, despite considerable size of the set studied.

1. The range of patients’ demographics is extremely broad. Such breadth is a result of initial randomising approach of the study, which was aimed at collecting data from a most representative population set. However, such an approach has as well drawback as advantages. Patients with quite different comorbidities entered the set, as there was no filtration of patients/carriers. In the study, the influence of comorbidities on COVID-19 clinical course was not studied quantitatively and thoroughly, as it may be a task for a separate investigation. Different comorbidities may lead to quite dissimilar COVID-19 clinical course and blur the VL/ARDS score/T cell count relationships. It was not paid due attention in the current work.
2. The main conclusion of the study is T cells counts’ being the best predictive markers for COVID-19 clinical course and mortality. Therefore, it may be implied that effective adaptive immune system response plays a major role in positive prognosis and immune impairments deteriorate the prognosis. However, we did not specifically and assiduously investigate patients with immunity disorders (we merely took into consideration the fact of clinically documented immunity diseases), nor immunity deterioration with age. As the set was very broad, many aged persons and people with impaired immunity were included in it, but their immunity functioning (e.g., median levels of T cell serum concentrations in the presence and absence of an acute infectious disease) before COVID-19 was not taken into account. This oversimplifying might distort the results to considerable degree.
3. After the extensive research, it is still unclear whether SARS-CoV-2 causes lymphopenia or, on the contrary, “cytokine storm” (hyperinflammation) as its most probable effect on immunity of a conditionally healthy adult. There are plenty of works supporting either assumption. The data obtained in our research cannot support a suggestion that SARS-CoV-2 causes any significant CD8^+^ and CD4^+^ lymphopenia itself, even less so suppression of other T lymphocyte populations. It is highly possible that SARS-CoV-2 may be an exacerbating factor of immune dysfunctions or diseases already present in people with serious disease- or age-related lymphocyte population decrease. E.g. HIV, HBV, HCV, immunosuppressed status after anti-cancer chemotherapy, organ/tissue transplantation/grafting etc. may cause substantial lymphopenia that SARS-CoV-2 may exaggerate further.
  We ground our assumption on the following observation. Of all deaths registered for patients with more than twofold T cell count diminution (either of T killer, Th1 helper, NKT cells, γδT cells or CD8αα^+^ cells), 87.4% were also associated with immune dysregulation/diseases not related to SARS-CoV-2 and clinically documented before the pandemic. Therefore, we suggest that the assumption about SARS-CoV-2’s resulting in immune dysregulation of a healthy person, should be re-evaluated on a more clinically checked set of SARS-CoV-2 carriers/patients, ideally in randomised controlled clinical trials.
  Primary targeting T killer and NKT cell populations instead of CD4^+^ lymphocyte population hints that SARS-CoV-2 influence on cell immunity is completely dissimilar with HIV-1. Indeed, it is possible that SARS-CoV-2 can act as an inductor or “amplifier” of immune disorders/dysfunction. If this is true, in most patients SARS-CoV-2 does not cause lymphopenia itself, rather only augments it, possibly like influenza or some other respiratory viruse (e.g. parainfluenza viruses)^47–53^.
  However, the role of SARS-CoV-2 in lymphopenia, whether it is primary or secondary, was not studied. Suppression of lymphocyte population size (lymphopenia) needs a further detailed research. We do not know biochemical mechanisms of such lymphopenia, i.e. whether they are related to membrane protein degradation or another distortion of T cell differentiation or activation.
4. SARS-CoV-2 was found to cause a wave-form correlation of leukocyte and plasma cell serum concentrations with VL in the majority of symptomatic patients and typical VLs of 10^5^–10^8^ RNA copies per reaction were determined to correspond with the maximum of the wave. This fact was not possible to interpret in the current study within any theory of immune response.
5. It is clear that SARS-CoV-2 targets adaptive immunity much more than innate immunity, and T and NKT cells more than γδT cells. However, homodimeric CD8αα^+^ lymphocyte population (a part of innate immunity) in pharyngeal MALT (and hence very likely in all parts of the upper respiratory tract MALT) is very seriously affected in severe COVID-19 cases and may be the best marker of a patient’s respiratory state deterioration. The finding has not yet been fully interpreted and comprehended in this research, partly because insufficiency of our data and partly because the role of CD8αα^+^ cells in human immune system is still not completely clear.
6. We had a rather arbitrary choice of measurement time of VL, PaO_2_/FiO_2_ ratio and leukocyte/lymphocyte counts, as specified in Methods section. No longitudinal studies were made in this work. The time of measurement was approximately the same for all carriers/patients that allowed to reduce the dispersion of results. However, we have to remember that dynamics of the parameters studied is very important, as these parameters change with COVID-19 clinical course and, according to the remark of our Reviewer, their alteration may indicate improvement or progression of COVID-19.
7. For the majority of patients, no immune tests have been ever made before the onset of COVID-19 disease (or detecting the asymptomatic course). We may only assume that their immune indicators (B and T cell counts) were within normal range of a conventionally healthy adult before COVID-19.

## Conclusions

1. T lymphocyte count may be a perspective marker of COVID-19 course severity and prognosis, more unambiguous than SARS-CoV-2 VL in the upper respiratory tract or respiratory index PaO_2_/FiO_2_. Using T cell count in clinical practice may provide an opportunity of early prediction of deteriorating a patient’s state.
2. A very broad range of SARS-CoV-2 VL (50–10^13^ RNA copies per reaction) may correspond to similar complications of COVID-19. That makes VL a poor indicator of COVID-19 clinical course severity.
3. Asymptomatic SARS-CoV-2 carriers, whose percentage in SARS-CoV-2-affected population was found to be large (more than two thirds of all infected persons), do not demonstrate any significant changes in the levels of B and T lymphocytes, while VLs in these carriers remain low.
4. The best marker of mortality was found to be a “complex factor” that can be calculated as a product of reversed logarithm of VL, T killer count, plasma cell count and NKT cell count in serum. The lowest values of this factor highlight extremely severe cases of COVID-19 disease with negative prognosis.
5. The best predictive marker of COVID-19-related severe ARDS was detected to be CD8αα^+^ lymphocyte count in pharyngeal MALT. Its sharp reduction may be used in clinical practice as an unambiguous indicator of necessity to admit a patient to ICU wards.

## Data Availability

Available upon a reasonable request.
